# Announcing *FEMS* Microbes: open science toward a sustainable world

**DOI:** 10.1093/femsmc/xtaa005

**Published:** 2020-09-23

**Authors:** Kimberly A Kline, Kathleen Scott, Jana Jass

**Affiliations:** Singapore Centre for Environmental Life Sciences Engineering and School of Biological Sciences, Nanyang Technological University, Singapore , 637819; Department of Integrative Biology, University of South Florida, Tampa, FL, USA, 33620; The Life Science Centre - Biology, School of Science and Technology, Örebro University, Örebro, Sweden , 70182

## INTRODUCTION

Microbes are a driving force in the ecology and evolution of life on Earth, and their place on center stage is becoming increasingly apparent. Climate change, environmental destruction and human encroachment into previously less populated locations have driven the expansion of microbial habitats and the opportunity for more frequent spillover events. Our ever-growing human population and medical interventions that increase lifespan, coupled with a highly interconnected world, amplify the possibilities for microbial expansion into niches and hosts where natural checks and balances on microbial growth are absent. Human activities also have a growing impact on global nitrogen and carbon cycles, mediated by microorganisms. Easy access to antimicrobials has been a salvation in many cases, but is also associated with the inevitable selection for antimicrobial-resistant microbes. Indeed in 2019, the WHO released the 10 greatest threats to global health (Table 1), 6 of which directly relate to microbial disease. These six threats include high-threat or high-prevalence viral pathogens, antimicrobial resistance (AMR) and vaccine hesitancy. The remaining four WHO health threats indirectly relate to microbes: climate change and fragile and vulnerable settings promote spillover events; noncommunicable diseases such as diabetes, cancer and heart disease serve as comorbidities that significantly increase likelihood for infection with poorer outcomes; and weak primary healthcare includes preventive care and treatment for infections. Research in the microbial sciences informs our approaches to preventing and combatting these current and future global health threats. Despite these challenges, the majority of the vastly diverse microbes are involved in global maintenance and environmental balance. Therefore, we not only greatly benefited from microbial activities but also learn from them and employ their strategies for our own benefit.

**Table 1.** WHO: 10 threats to global health in 2019 (WHO 2019).

Global influenza pandemic	Directly microbial related
Antimicrobial resistance	Directly microbial related
Ebola and other high-threat pathogens	Directly microbial related
Vaccine hesitancy	Directly microbial related
Dengue	Directly microbial related
HIV	Directly microbial related
Air pollution and climate change	Indirectly microbial related
Noncommunicable disease	Indirectly microbial related
Fragile and vulnerable settings	Indirectly microbial related
Weak primary healthcare	Indirectly microbial related

### One Health from a microbial vantage point

The One Health paradigm operates at the intersection of human health, ecosystem health and animal health. As defined by the US CDC, One Health is ‘… a collaborative, multisectoral, and transdisciplinary approach—working at the local, regional, national, and global levels—with the goal of achieving optimal health outcomes recognizing the interconnection between people, animals, plants, and their shared environment.’ (CDC 2018). At FEMS Microbes, we see microbes acting at each level and interface within the One Health framework, and we use this paradigm as an organizational structure for the journal.

### Microbial sciences and the United Nations sustainable development goals

Microbiologists work to understand, harness and control microbes for a better world. The UN sustainable development goals (SDGs) serve as a ‘… blueprint to achieve a better and more sustainable future for all. They address the global challenges we face, including those related to poverty, inequality, climate, environmental degradation, prosperity, and peace and justice. The Goals interconnect and … leave no one behind …’ (UN 2015). Microbial problems are integral to many of these global challenges. Microbiologists can therefore serve as key drivers in the solutions and implementation of many of the SDGs. The role for microorganisms in each SDG has been considered (Akinsemolu 2018); we highlight here those in which microbes have an outsized role.

Goal 2: Zero Hunger, aims to end hunger, achieve food security and improved nutrition, and promote sustainable agriculture. Microbial and related synthetic biology disciplines are poised to optimize food production, nutrition and safety—enabling delivery of high quality, low-cost food to those who need it most.

Goal 3: Good Health and Well-Being, seeks to ensure healthy lives and promote well-being for all at all ages. Controlling infectious disease through better therapeutics, developing vaccines for existing and emerging health threats, including AMR, promoting beneficial microbiomes and appreciating the molecular epidemiology of microbes will help achieve this goal. It is increasingly apparent that our individual microbiome has great impact on our health and well-being.

Goal 4: Clean Water and Sanitation, works to ensure availability and sustainable management of water and sanitation for all. An increased understanding of microbial communities and their functions in wastewater processing and bioremediation will optimize the ability of communities to provide clean and safe water supply pipelines.

Goal 7: Affordable and Clean Energy, aims to ensure access to affordable, reliable, sustainable and modern energy for all. Microbial biofuels and microbial fuel cells are attractive sources for renewable energy, producing energy from existing organic matter, if they can be scaled up to levels required for practical power production.

Goal 9: Industry, Innovation and Infrastructure, seeks to build resilient infrastructure, promote inclusive and sustainable industrialization and foster innovation. Microbes can be harnessed for the synthesis of a variety of industrial chemicals and polymers, including new drugs and antimicrobials, as well as for their decomposition activities important for bioremediation.

Goal 14: Life Below Water, strives to conserve and sustainably use the oceans, seas and marine resources for sustainable development. Healthy marine ecosystems depend on a healthy microbial community. One need look no further for evidence than the massive coral bleaching resulting from algae loss, rendering coral more susceptible to death. Safe and productive aquaculture also relies on healthy fish microbiomes and the ability to resist colonization by fish pathogens. There is considerable interest in environmentally safe approaches controlling biofouling by microbial biofilms of ship hulls, which is a major cost for shipping and military industries.

Goal 15: Life on Land, seeks to protect, restore and promote sustainable use of terrestrial ecosystems, sustainably manage forests, combat desertification, and halt and reverse land degradation and halt biodiversity loss. The terrestrial biosphere consists of a diverse yet important microbial interface between abiotic substances and living organisms. The routine use of antibiotics in healthy farm animals has contributed to the AMR crisis; safe alternatives for farmers' industries are essential to preserve remaining effective antibiotics. Understanding the microbial diversity on land serves many purposes, but most prominently in current times, it enables our ability to rapidly trace the origins of emerging pathogens in animals and humans, such as SARS-COV-2.

In addition to SDGs that directly relate to microbes, others indirectly intersect with the goals of FEMS and FEMS Microbes. In particular, Goal 3: Quality Education and Goal 5: Gender Equality are key priorities for us.

### The new FEMS Microbes journal

As an open access journal, FEMS Microbes makes the latest in microbial science research readily and freely available to all. FEMS Microbes also prioritizes inclusivity and diversity and we aim to both represent and harness the capacity of all the microbiology community around the world. To achieve this, we encourage submissions from early career scientists and will provide special opportunities to highlight the work of these individuals. Our editorial board reflects this commitment in both gender and geographic diversity, and aims to reflect this same diversity during the peer review process by drawing on reviewers from all career stages and from around the world.

FEMS Microbes provides a venue for the sharing of microbial research that tackles the biggest challenges of our lifetime head-on, ranging from fundamental research that underpins our basic understanding of microbes to translational studies that aim to solve these challenges. FEMS Microbes will publish high-quality basic and applied research within diverse areas of industrial, environmental, clinical and veterinary microbiology, as well as current methodological advancements that are of general interest. The journal will be organized according to the following themes:

**Microbes & Metabolism**. A fundamental understanding of microbial processes at the genetic, biochemical and metabolic levels is necessary for harnessing and controlling microbes to address threats to global health.
**Microbes & Multicellular Life**. Microbial biofilms and communities serve many functions in our biological world, including promoting the health and development of plants and animals, and serving to keep invading microbes in check.
**Microbes & Disease**. Population expansion, climate change, deforestation, mismanagement of antimicrobial usage and other means of environmental damage lead to increased contact with animal vectors leading to new and re-emerging infectious diseases including zoonoses, vector-borne diseases and expansion of AMR.
**Microbes & Environment**. Changes in microbial ecology go hand in hand with changes in the environment and in animals. Microbial ecology drives ecosystem health, agricultural practices, waste management, bioremediation and the often precarious balance between health and disease.
**Microbes & Society**. The activity of microorganisms impacts humanity in a myriad of ways, ranging from food safety and production to clean water supplies to development of new therapeutics. Many biotechnological advances have direct or indirect underlying microbial origins that are exploited.

## CONCLUSION

It is apparent that we are highly dependent on the microbial world around and within us, resulting in both detrimental and beneficial consequences. A better understanding of microbes will help us navigate this interdependent relationship, with the aim of solving some of our major problems and improving life. From this perspective, FEMS Microbes is an exciting new journal that aims to facilitate rapid and open knowledge transfer within the microbiology community on all aspects associated with microbes.
